# Clinical emergence of a novel sequence type (ST3672) NDM-1-producing *Vibrio parahaemolyticus* in foodborne disease

**DOI:** 10.3389/fmicb.2026.1767946

**Published:** 2026-04-22

**Authors:** Chengpei Ni, Jiancheng Wang, Chao Yang, Song Gao, Ze Xu, Yajing Wang, Honghong Peng, Wengting Cao, Yi Han

**Affiliations:** 1 The Affiliated Wuxi Center for Disease Control and Prevention of Nanjing Medical University, Wuxi Center for Disease Control and Prevention, Wuxi, China; 2 Shanghai Institute of Immunology and Infection, Chinese Academy of Science, Shanghai, China

**Keywords:** antibiotic resistance, IncC plasmid, NDM-1, *Vibrio parahaemolyticus*, virulence factors

## Abstract

**Background:**

This study characterized a newly identified ST3672-type *Vibrio parahaemolyticus* (*V. parahaemolyticus*) strain (VP1434), isolated directly from a patient involved in a food poisoning outbreak, highlighting its rare multidrug resistance (MDR) phenotype. The objectives were to investigate its resistance mechanisms, elucidate its evolutionary relationships, and assess its pathogenicity.

**Methods:**

The VP1434 isolate was analyzed as follows: (1) Antimicrobial susceptibility was tested against 29 antibiotics, and plasmid conjugation experiments verified drug resistance in recipient strains post-transfer. (2) Whole-genome sequencing was used to characterize its genomic features, including plasmid sequences and inter-plasmid relationships. (3) Evolutionary tracing of VP1434 and the plasmid pVP1434-NDM was performed by constructing phylogenetic trees. (4) Virulence factors (VFs) were identified via the Virulence Factor Database, and pathogenicity was assessed through mouse infection.

**Results:**

The strain demonstrated a concerning MDR profile, exhibiting resistance to all tested *β*-lactam antibiotics. Additionally, it displayed resistance to sulfonamides and aminoglycosides, with intermediate susceptibility to tetracyclines. Plasmid conjugation experiments showed the *bla*_NDM-1_-harboring plasmid was transferable to VP0577, and transconjugants also showed MDR. Whole-genome sequencing identified a *bla*_NDM-1_-carrying plasmid harboring multiple resistance genes, which fully explaining its phenotypic resistance to at least four classes of antibiotics. Phylogenetic analysis placed VP1434 on a distinct branch within the maximum-likelihood tree constructed from global *V. parahaemolyticus* isolates, while the closest relative of the *bla_NDM-1_*-harboring plasmid was identified in a shrimp-derived *V. parahaemolyticus* strain. VP1434 was found to harbor 185 VFs, including *tlh* and *trh*, but lacked the key pathogenicity determinant *tdh*. In mouse infection experiments, strain VP1434 (*tdh^−^/trh^+^*) exhibited significantly higher lethality than environmental isolates (*tdh^−^/trh^−^*), suggesting TRH plays a significant role in pathogenicity even in the absence of TDH.

**Conclusion:**

This first report of *bla*_NDM-1_-positive clinical *V. parahaemolyticus* reveals plasmid-mediated MDR emergence in human infections. Its unexpected virulence without classical toxins indicates evolutionary adaptation, necessitating diagnostic updates and strengthened surveillance.

## 1 Introduction

*Vibrio parahaemolyticus* (*V. parahaemolyticus*) is a significant foodborne pathogen causing gastroenteritis, wound infections, and septicemia (Yang et al., 2022). Pathogenic strains of *V. parahaemolyticus* are capable of inducing gastrointestinal disorders in humans, exemplified by acute gastroenteritis that arises following the ingestion of seafood contaminated with the bacterium, particularly through the consumption of inadequately cooked or uncooked shellfish and fish. The virulence of *V. parahaemolyticus* is predominantly attributed to hemolysin proteins, which are encoded by the genes responsible for thermostable direct hemolysin (*tdh*) and TDH-related hemolysin (*trh*) genes (Zin et al., 2025). These pore-forming toxins damage intestinal cell membranes, causing lysis, inflammation, and characteristic symptoms including diarrhea and cramping. In addition, the bacterium also encodes for adhesions and type III secretion systems (T3SS1 and T3SS2) to ensure its survival in the environment. While these virulence genes aren’t ubiquitous among strains, their presence correlates strongly with increased pathogenicity and disease severity (Ong et al., 2023).

Of growing concern is the increasing antibiotic resistance exhibited by *V. parahaemolyticus* in aquaculture, compounding its public health threat alongside rising infection rates (Tan et al., 2022; Xu et al., 2024). The widespread use of antimicrobials in humans and animals has contributed to increasing antimicrobial resistance (AMR). *V. parahaemolyticus* infections may progress to bloodstream invasion (septicaemia) in severe cases. For managing persistent or systemic infections, clinicians typically employ antimicrobial therapy including fluoroquinolones, macrolides (e.g., azithromycin), or the non-absorbable antibiotic rifaximin. Wild-type *V. parahaemolyticus* strains generally demonstrate sensitivity to most antimicrobial categories, with the notable exception of penicillin-class drugs (Briet et al., 2018). Aquaculture farmers frequently employ widespread prophylactic antibiotics in farmed fish to combat *V. parahaemolyticus* and its biofilms, a strategy that has sparked grave concerns over the rising antibiotic resistance in this bacterium (She et al., 2023). While many antimicrobial-resistant *V. parahaemolyticus* strains have been isolated from seafood (Briet et al., 2018; Oyelade et al., 2018), reports of multidrug-resistant (MDR) clinical isolates remain rare. The isolation of MDR strains from clinical patients would pose substantial challenges for clinical management (Fang et al., 2023; Bai et al., 2025).

New Delhi metallo-*β*-lactamase-1 (*bla*_NDM-1_) is a clinically important carbapenemase that confers broad beta-lactam resistance. NDM-1 gene-carrying bacteria represent an emerging class of drug-resistant “superbugs” capable of breaking down nearly all β-lactam class antibiotics. These microorganisms demonstrate remarkable transmission capabilities, spreading rapidly both within their own species and across different bacterial species (Wang et al., 2021). Their adaptable enzymatic degradation mechanism, coupled with the continuous emergence of new variants, significantly worsens the challenge of antimicrobial resistance. Although aztreonam (a monobactam) inhibits NDM-1, no broad-spectrum clinically applicable inhibitors are available, posing severe risks to individual health and global public health security. The *bla*_NDM-1_ gene is primarily carried by five incompatibility (Inc) groups of plasmids: IncC, IncW, IncpPROV114-NR, IncpCHS4.1–3, and IncpPrY2001 (Wang et al., 2023). Among these, IncC plasmids are particularly efficient at disseminating resistance due to their high stability, broad host range, and conserved conjugation system (Nagulapalli Venkata et al., 2021; Fang et al., 2024). Although *V. parahaemolyticus* isolates carrying the *bla*_NDM-1_ gene have been increasingly reported in food and aquatic products over the past 2 years, *bla*_NDM-1_ -positive strains recovered from human clinical specimens remain undocumented (Chen et al., 2025). Furthermore, marked disparities exist between environmental and clinical pathogenic isolates of this species. Our investigation identified a multidrug-resistant (MDR) strain of *V. parahaemolyticus*, which was isolated from anal swabs of patients involved in a foodborne outbreak and found to carry an IncC-type *bla*_NDM-1_plasmid. The strain’s resistance phenotype and genomic characteristics warrant thorough investigation to elucidate its emergence mechanisms, transmission risks, and public health implications. The emergence of *bla*_NDM-1_ plasmid-carrying bacteria isolated from human samples underscores the need for prioritized surveillance, as it poses a critical threat to public health security through the potential spread of multidrug resistance.

## 2 Materials and methods

### 2.1 Strain isolation and cultivation

The *V. parahaemolyticus* strain VP1434 was isolated from the anal swab specimens of patients involved in a food poisoning incident that occurred on July 21, 2024, in Wuxi City, Jiangsu Province, China. This strain was identified as a member of the genus *Vibrio* based on its phenotype on thiosulfate-citrate-bile salts-sucrose (TCBS) agar plates and incubated at 37 °C for 18–24 h. At least three typical colonies of *V. parahaemolyticus* were isolated from each plate and subjected to identification by multiplex PCR assays and DNA sequencing. The genetic identity of the isolates was further confirmed by the Vitek 2™ System and MALDI-TOF MS. Biochemical identification was performed using the VITEK® 2 Gram-Negative Identification Card (GN). The reagent composition is provided in the (Supplementary Appendix Table S1). The isolated strains were preserved as frozen cultures in 20% glycerol at −80 °C, and Alkaline Peptone Water with 3% NaCl or agar was used for routine culture.

### 2.2 Antimicrobial susceptibility tests

Antimicrobial susceptibility tests were conducted on the isolates, employing the standard agar dilution technique specified by the Clinical and Laboratory Standards Institute (CLSI). To ensure quality control, the *Escherichia coli* strain ATCC 25922 was incorporated. Antimicrobial susceptibility testing of VP1434 was performed using a panel of 29 clinically relevant antibiotics (Supplementary Appendix Table S2). Based on CLSI antimicrobial susceptibility criteria, the outcomes were categorized as sensitive (S), intermediate (I), or resistant (R).

### 2.3 Conjugation assay

Conjugation was performed as described in previous studies (Couturier et al., 2023). The recipient strain was *V. parahaemolyticus* RIMD WT harboring a plasmid (VP0577, SpcR) with the pRP1028 backbone, which was constructed as previously reported (Yang et al., 2025), and VP1434 was used as the donor strain. Overnight cultures of the donor and recipient strains were subcultured in antibiotic-free LB medium supplemented with 3% NaCl (mLB) and grown to an OD₆₀₀ of 0.5–0.6. The cultures were harvested by centrifugation, the supernatant was discarded, and the cell pellets were washed and resuspended in the same antibiotic-free mLB medium. Equal volumes (500 μL each) of the donor and recipient suspensions were mixed at a 1:1 ratio and incubated statically at 37 °C for 4 h. The conjugation mixture was serially diluted and spread onto TSB agar plates supplemented with cefotaxime (8 μg/mL) and spectinomycin (50 μg/mL) for transconjugant selection, with donor and recipient strains processed in parallel as controls. Presumptive transconjugants were verified by antimicrobial susceptibility testing and PCR targeting both resistance genes and *V. parahaemolyticus* virulence genes.

### 2.4 Whole-genome sequencing and Bioinformatic analysis

The newly sequenced strains underwent whole-genome sequencing on the Illumina NovaSeq and Oxford Nanopore platforms. Raw reads were assembled *de novo* using the Unicycler v0.5.1 pipeline (https://github.com/rrwick/Unicycler) after removing low-quality sequences. Acquired antimicrobial resistance genes and plasmid identification were determined using the Center for Genomic Epidemiology’s ResFinder and PlasmidFinder tools, respectively. Multi-locus sequence typing (MLST) was conducted via the mlst tool (https://github.com/tseemann/mlst), referencing the PubMLST databases. The VFDB (Virulence Factor Database) was utilized as a reference database for conducting BLAST alignment to systematically identify and functionally annotate bacterial virulence factors. Prokka was employed for genome annotation and verification of gene cluster.

### 2.5 Phylogenetic analysis

The dataset comprised 8,685 global *V. parahaemolyticus* isolates, including strain VP1434. A maximum-likelihood phylogeny was then constructed based on their core-genome SNPs. Publicly available genomes were obtained from NCBI GenBank/SRA, with associated metadata extracted from BioSample records or published literature (Completeness > 90%, contamination < 5%). The construction method was based on our previously published research, with certain adjustments made. Briefly, genome sequences were aligned to a reference strain using MUMmer v3.23, followed by core SNP identification (present in > 99% of isolates) with SNP-sites v2.5.1 after masking repetitive regions (filtered using TRF v4.07b and BLASTN). Recombination regions were detected with Gubbins v3.1.352, and homoplasic SNPs were identified using SNPPar v1.053. Both recombined and homoplasic SNPs were excluded from the phylogenetic analysis. Phylogenetic reconstruction was performed with FastTree v2.1.10 on the filtered SNP set, and the resulting tree was visualized and annotated in iTOL. For the phylogenetic tree construction of NDM-1-encoding plasmids, the dataset comprised plasmids carrying the NDM-1 gene, retrieved via BLAST search using the complete nucleotide sequence of plasmid pVP1434-NDM. All *Vibrio*-derived plasmids were retained, while only one plasmid with the smallest E-value was included for each other bacterial species. Phylogenetic tree construction was performed as follows: selected plasmid sequences were aligned using MAFFT (v7.520), followed by trimming of the multiple sequence alignment results with trimAl (v1.5.rev1). A maximum-likelihood phylogenetic tree was subsequently constructed using RAxML-NG (v1.2.2).

### 2.6 Mouse lethality assay

SPF-grade BALB/c mice were purchased from Spefco (Suzhou) Biotechnology Co., Ltd. The mice were housed under specific conditions with a constant temperature of 22 ± 2 °C, a constant relative humidity of 50 ± 10%, and a 12 h light /12 h dark cycle, with free access to food and water. All mice were intraperitoneally inoculated with *V. parahaemolyticus* in a single dose, and then observed every 3 h to record their mental status, disease manifestations and other related indicators until the end of the experiment. Female BALB/c mice (4–5 weeks old) were randomly allocated into three groups (*n* = 10 per group) and intraperitoneally challenged with distinct *V. parahaemolyticus* strains. (1) VP0016: An environmental ST3041 strain lacking *tdh* and *trh* virulence genes. (2) VP0005: A clinical pathogenic ST3 strain harboring both *tdh* and *trh* virulence genes. (3) VP1434: A clinical ST3672 isolate (from this study) lacking *tdh* and harboring *trh* virulence genes. Bacterial cultures were harvested at the logarithmic phase, washed three times with phosphate-buffered saline (PBS), and resuspended in PBS. Mice were inoculated intraperitoneally with 4.5 × 10^7^ CFU of the respective strain suspension, and mortality was recorded at predetermined time points. All animal procedures, including colonization and lethality assays, were approved by the Animal Ethics Committee of Nanjing Medical University. Survival curves were generated using the Kaplan–Meier method, and statistical differences between groups were assessed by the log-rank test. A *p*-value < 0.05 was considered statistically significant. *: *p* < 0.05, **: *p* < 0.01; ***: *p* < 0.001.

### 2.7 Nucleotide sequence accession numbers

The sequence data of VP1434 has been deposited in the National Microbiology Data Center (NMDC) under accession numbers NMDC60213730^1^ and in NCBI GenBank under accession number JBVYWM000000000.^2^

## 3 Results

### 3.1 Patient phenotypes and strain biochemicals

On July 21, 2024, a food poisoning outbreak occurred in Wuxi City, Jiangsu Province, China, affecting five individuals. The index case developed symptoms at 7:30 on July 22, presenting with nausea, vomiting (5 episodes/day), episodic upper abdominal pain, profuse watery diarrhea (≥10 episodes/day), and dizziness. All specimens were collected by the local Center for Disease Control and Prevention (CDC) prior to any clinical treatment after the patients were collectively hospitalized. From this patient, a *V. parahaemolyticus* strain (VP1434) was isolated and identified through 16S rRNA sequence analysis, biochemical tests (Table 1) and MALDI-TOF MS analysis (data not shown). All patients achieved complete recovery and were discharged uneventfully following symptomatic fluid replacement therapy and targeted antimicrobial treatment with levofloxacin, a first-line quinolone agent for the clinical management of *Vibrio*-associated foodborne diseases, as prescribed by attending physicians based on clinical judgment; no fatalities occurred in this incident.

**Table 1:** Biochemical details of the isolate from VITEK-2.

No.	Test name	Results	No.	Test name	Results	No.	Test name	Results
1	APPA	+	17	PyrA	+	33	dCEL	−
2	H2S	−	18	AGLTp	−	34	GGT	−
3	BGLU	+	19	dMAN	+	35	BXYL	−
4	ProA	+	20	PLE	−	36	URE	+
5	SAC	−	21	dTRE	+	37	MNT	−
6	lLATk	+	22	SUCT	+	38	NAGA	−
7	GlyA	+	23	LDC	−	39	CMT	+
8	O129R	+	24	lMLTa	−	40	lLATa	−
9	ADO	−	25	lARL	−	41	BGAL	−
10	BNAG	+	26	dGLU	+	42	OFF	+
11	dMAL	+	27	dMNE	+	43	BAlap	−
12	LIP	−	28	TyrA	+	44	dSOR	−
13	dTAG	−	29	CIT	−	45	5KG	−
14	AGLU	−	30	AGAL	−	46	PHOS	−
15	ODC	−	31	lHISa	−	47	BGUR	−
16	GGAA	+	32	ELLM	−			

Biochemical test abbreviations follow the VITEK-2 system (bio Mérieux, France).

**+**, Positive; **−**, Negative.

Biochemical test results (Table 1) demonstrated that strain VP1434 exhibited the characteristic biochemical profiles of *V. parahaemolyticus*. Specifically, it was Gram-negative and unable to ferment sucrose (5, SAC), while capable of fermenting glucose (42, OFF), D-mannitol (19, dMAN), and D-maltose (11, dMAL), with no hydrogen sulfide (2, H₂S) gas production observed. Notably, this strain was negative for both lysine decarboxylase (23, LDC) and ornithine decarboxylase (15, ODC)—a phenotype rarely reported in *V. parahaemolyticus.* Prokka-based genome annotation demonstrated that the *cadA* and *cadB* genes are present in VP1434, which encode lysine decarboxylase and ornithine decarboxylase, respectively. As reported previously, environmental isolates of this species generally exhibit positive reactions for lysine decarboxylase and ornithine decarboxylase, while a proportion of clinical pathogenic strains test negative for these enzymes. The enzymatic activities of these proteins are likely to be more readily expressed *in vivo* (Jones et al., 2012). Furthermore, VP1434 tested positive for urease (36, URE). Subsequent genomic analysis using Prokka confirmed the presence of a complete urease gene cluster (*ureA, ureB, ureC, ureD, ureE, ureF, ureG, and ureR*) in its genome. Urease, encoded by this cluster, is widely recognized as a key virulence factor in clinical isolates, as it facilitates ammonia production during bacterial infection, leading to intestinal fluid accumulation and gastrointestinal inflammation (Wang et al., 2015).

### 3.2 Antibiotic resistance phenotypes and genetic determinants

Antimicrobial susceptibility testing of VP1434 was performed using a panel of 29 clinically relevant antibiotics (Supplementary Appendix Table S2). The strain exhibited a concerning multidrug-resistant (MDR) profile, demonstrating complete resistance (R) to all tested *β*-lactam antibiotics including penicillins (ampicillin MIC > 64 μg/mL, ampicillin/sulbactam MIC > 64 μg/mL), β-lactam/β-lactamase inhibitor combinations (amoxicillin/ clavulanate MIC = 32 μg/mL), and cephalosporins across all generations (cefazolin MIC > 32 μg/mL [first generation], cefuroxime MIC > 64 μg/mL and cefoxitin MIC = 64 μg/mL [second generation], ceftazidime MIC > 32 μg/mL and cefotaxime MIC > 8 μg/mL [third generation], cefepime MIC > 8 μg/mL [fourth generation]). Additionally, it showed resistance to sulfonamides (compound sulfamethoxazole MIC > 8 μg/mL) and aminoglycosides (streptomycin MIC > 32 μg/mL). An intermediate (I) phenotype was observed for tetracycline (MIC = 8 μg/mL), indicating emerging reduced susceptibility to this class of antibiotics (Table 2). To investigate the resistance mechanisms, whole-genome sequencing (Illumina/Nanopore) was performed, identifying two chromosomes and a plasmid carrying the *bla*_NDM-1_ gene, which was accordingly designated pVP1434-NDM. ResFinder analysis confirmed that pVP1434-NDM carries multiple resistance genes, including *bla*_NDM-1_*, bla*_CARB-44_*, sul1, sul2, tetA, aph(6)-Id, and aph(3″)-Ib*. Additionally, *floR* and *dfrA10* were detected, conferring predicted resistance to chloramphenicol/florfenicol and trimethoprim, respectively. The strain’s resistance profile correlated closely with this *bla*_NDM-1_-positive plasmid. Both genotypic and phenotypic analyses confirmed that VP1434 is an MDR strain, exhibiting resistance to over three classes of antibiotics, namely *β*-lactams, folate pathway inhibitors, tetracyclines, and aminoglycosides.

**Table 2:** Drug-resistant phenotype and genetic determinants in *V. parahaemolyticus* strain VP1434.

No.	Antibiotic	MIC (ug/ml)	Phenotype	Genetic element	Location
1	Ampicillin	>64	R	*bla*_CARB-44_	chromosome
				*bla*_NDM-1_	plasmid
2	Ampicillin/Sulbactam	>64	R	*bla*_NDM-1_	plasmid
3	Amoxicillin/Clavulanate	32	R	*bla*_NDM-1_	plasmid
4	Ceftazidime	>32	R	*bla*_NDM-1_	plasmid
5	Cefotaxime	>8	R	*bla*_NDM-1_	plasmid
6	Cefoxitin	64	R	*bla*_NDM-1_	plasmid
7	Cefepime	>8	R	*bla*_NDM-1_	plasmid
8	Cefuroxime	>64	R	*bla*_NDM-1_	plasmid
9	Cefazolin	>32	R	*bla*_NDM-1_	plasmid
10	Compound sulfonamides	>8	R	*sul1,sul2*	plasmid
11	Streptomycin	>32	R	*aph(6)-Id,aph(3″)-Ib*	plasmid
12	Tetracycline	8	I	*tet(A)*	plasmid
13	Chloramphenicol	4	S	*floR*	plasmid
14	Trimethoprim	ND	ND	*dfrA10*	plasmid

MIC indicates Minimum inhibitory concentration (μg/mL). MICs were determined by broth microdilution following CLSI M45. Breakpoints were applied per CLSI M100-S32 for Vibrio spp. ND indicates not determined (phenotypic resistance test not performed). Resistance categories: R (Resistant), I (Intermediate), and S (Susceptible). The susceptibility/resistance determination standards for strains to various antibacterial agents in this table refer to the standards of the U.S. Food and Drug Administration (FDA), the European Committee on Antimicrobial Susceptibility Testing (EUCAST), and the relevant documents of the Clinical and Laboratory Standards Institute (CLSI): M100-S32, M45-A3, and VET01-A4.

### 3.3 Results of plasmid conjugative transfer

To ascertain whether the multidrug resistance in VP1434 is mediated by the *bla*_NDM-1_ -carrying plasmid, we performed a conjugation assay using VP1434 (donor) and VP0577 (recipient). VP0577 is a genetically modified derivative of a clinical *V. parahaemolyticus* isolate that retains the *tdh* virulence gene and harbors spectinomycin resistance. This engineered strain allows rigorous assessment of the transfer risk of the *bla*_NDM-1_ carrying plasmid into hypervirulent *V. parahaemolyticus* backgrounds. Based on the conjugation formula, the plasmid transfer frequency was determined to be 1.04 ± 0.17 × 10^−3^. Subsequently, we evaluated the MIC values of the strains obtained through conjugation named VP1434-VP0577. The conjugant strain VP1434-VP0577 demonstrated robust resistance to cephalosporins, mirroring the resistance profile of VP1434 (Table 3). It exhibited resistance to ampicillin, ampicillin/sulbactam, amoxicillin/clavulanate, ceftazidime, cefotaxime, cefoxitin, cefepime, cefuroxime, cefazolin, compound sulfonamides, and chloramphenicol. However, a slight reduction in MIC values was noted for ampicillin and cefoxitin, with values decreasing from 64 μg/mL to 32 μg/mL. Additionally, the MIC values for tetracycline and chloramphenicol also decreased by one dilution gradient; tetracycline shifted from intermediate to susceptible (MIC reduced from 8 μg/mL to 4 μg/mL), whereas chloramphenicol remained resistant (MIC decreased from 4 μg/mL to 2 μg/mL). Although the recipient strain VP0577 in itself displayed resistance to cefazolin with an MIC of 8 μg/mL, the MIC for the conjugant strain VP1434-VP0577 increased to greater than 32 μg/mL, indicating that the *bla*_NDM-1_ plasmid significantly enhanced its resistance. Furthermore, VP1434-VP0577, similar to VP0577, exhibited resistance to spectinomycin.

**Table 3:** Antibiotic susceptibility and minimum inhibitory concentrations of *V. parahaemolyticus* strains.

No	Antibiotic	VP1434		VP0577		VP1434—VP0577	
		MIC (ug/ml)	Phenotype	MIC (ug/ml)	Phenotype	MIC (ug/ml)	Phenotype
1	Ampicillin	>64	R	4	S	32	R
2	Ampicillin/Sulbactam	>64	R	≤1	S	>64	R
3	Amoxicillin/Clavulanate	32	R	2	S	32	R
4	Ceftazidime	>32	R	≤0.5	S	>32	R
5	Cefotaxime	>8	R	≤0.12	S	>8	R
6	Cefoxitin	64	R	4	S	32	R
7	Cefepime	>8	R	≤0.5	S	>8	R
8	Cefuroxime	>64	R	4	S	>64	R
9	Cefazolin	>32	R	8	R	>32	R
10	Compound sulfonamides	>8	R	≤0.25	S	>8	R
11	Streptomycin	>32	R	8	S	>32	R
12	Tetracycline	8	I	≤1	S	4	S
13	Chloramphenicol	4	S	8	S	2	S
14	Spectinomycin	≤2	S	>128	R	>128	R

MIC indicates Minimum inhibitory concentration (μg/mL). Resistance categories: R (Resistant), I (Intermediate), and S (Susceptible). The susceptibility/resistance determination standards for strains to various antibacterial agents in this table refer to the standards of the U.S. Food and Drug Administration (FDA), the European Committee on Antimicrobial Susceptibility Testing (EUCAST), and the relevant documents of the Clinical and Laboratory Standards Institute (CLSI): M100-S32, M45-A3, and VET01-A4.

Collectively, these results confirm that the *bla*_NDM-1_ carrying plasmid is a pivotal factor contributing to the cephalosporin resistance in VP1434 and demonstrate its high transfer efficiency.

### 3.4 Features of the pVP1434-NDM plasmid

**Figure 1:**
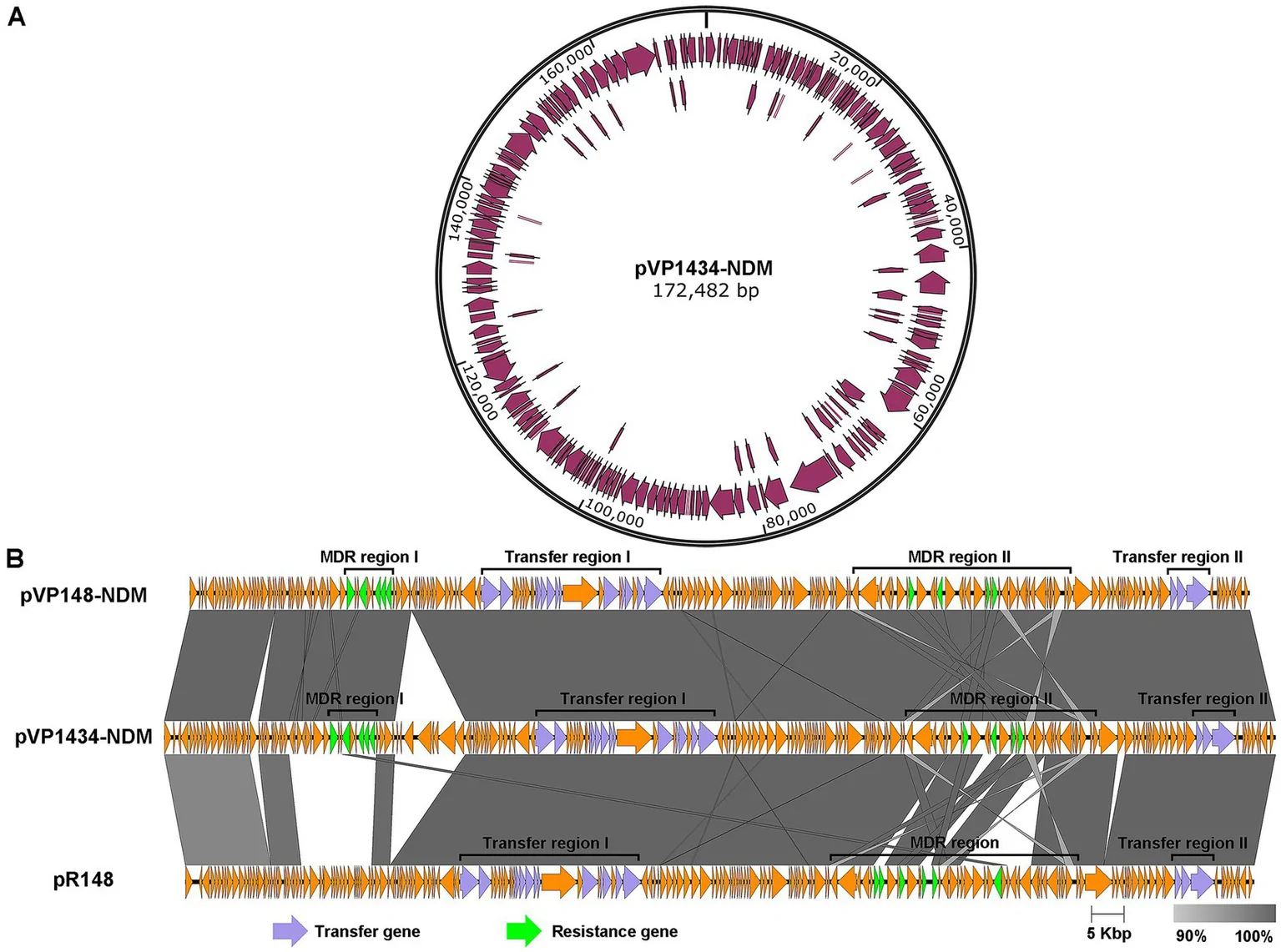
**(A)** Plasmid map of pVP1434-NDM. **(B)** The linear sequence comparison of pVP1434-NDM with two closely related plasmids (pR148 and pVP148-NDM).

Analysis of the genomic sequence information of the plasmid obtained through sequencing revealed that the complete nucleotide sequence of plasmid pVP1434-NDM, isolated from strain VP1434, is 172,482 base pairs (bp) in length, has an average G + C content of 52.15%, and comprises 176 predicted coding sequences (CDSs) (Figure 1A). BLAST analysis revealed that the backbone of pVP1434-NDM, shared an extremely high degree of genetic similarity (query coverage 93%) with a typical type 1a IncC plasmid, namely pVP148-NDM (accession no: PP860830), which was previously recovered from a *V. parahaemolyticus* isolate derived from shrimp (Figure 1B) (Zheng et al., 2019). The linear sequence comparison revealed that pVP1434-NDM possesses additional mobile elements compared to pVP148-NDM. After removing the additional mobile elements and MDR region I, the backbones of these two plasmids exhibited a high degree of similarity with pR148 (accession no: JX141473), a recognized type 1a IncC plasmid recovered from *Aeromonas hydrophila* in Thailand (Del Castillo et al., 2013). Plasmids pVP148-NDM and pVP1434-NDM exhibited > 99.9% nucleotide identity to plasmid pR148, with alignment coverage of 89 and 84% of their full-length sequences, respectively. The pVP1434-NDM plasmid may share key characteristics with IncC-type plasmids, including efficient conjugative transfer capability, a broad host range, and the carriage of multidrug resistance genes (Wu et al., 2019).

### 3.5 Phylogenetic analysis of *bla*_NDM-1_ carrying plasmids

**Figure 2:**
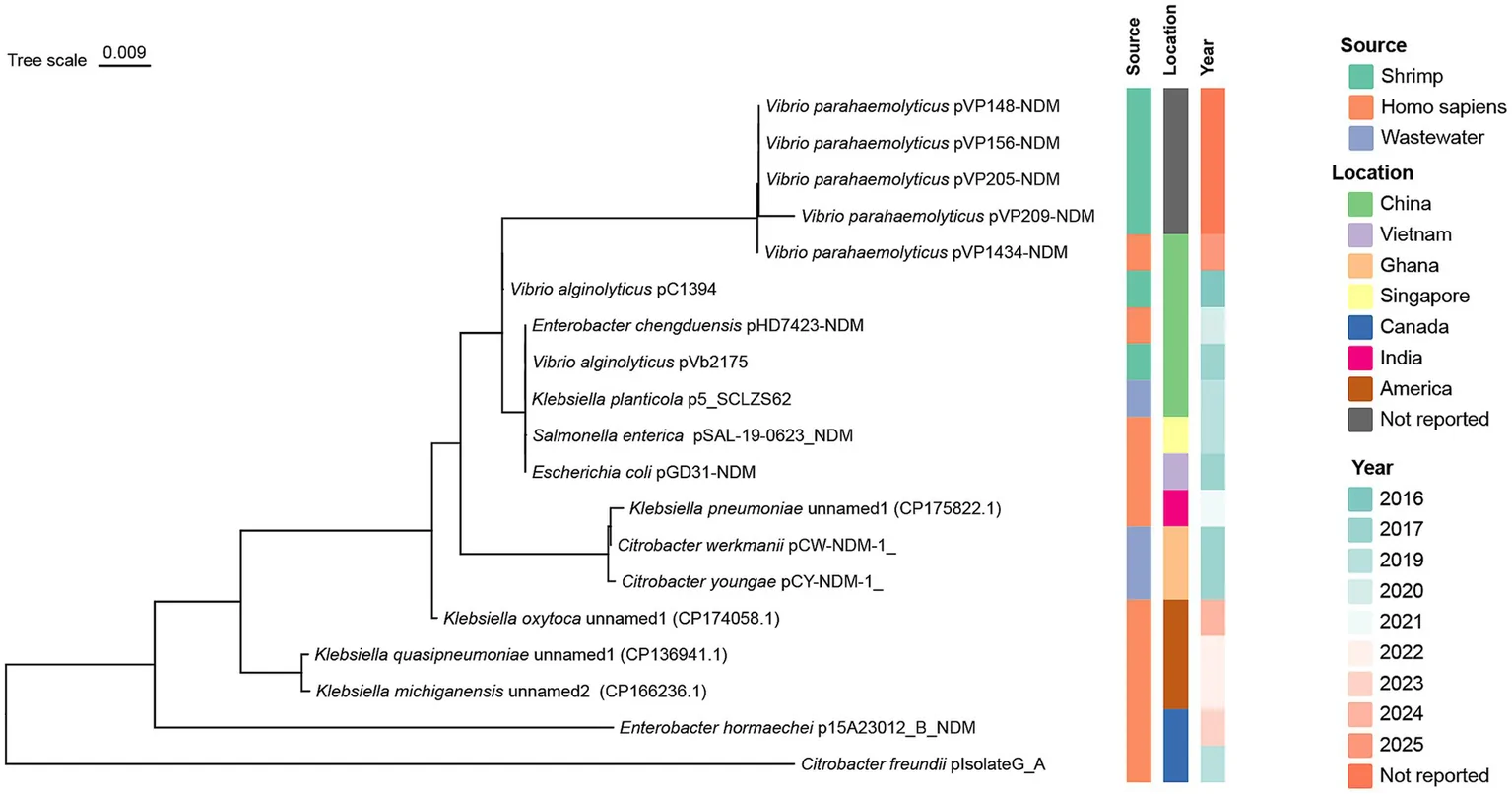
Phylogenetic tree of pVP1434-NDM (from VP1434) and other 18 NDM-1-harboring plasmids. All plasmid sequences were retrieved from the NCBI public database on February 20, 2026. Sequences obtained from the NCBI GenBank database are indicated by their names; uncharacterized sequences are shown with their accession numbers in parentheses. The tree was constructed using the maximum-likelihood method, with a scale bar of 0.009 indicating the evolutionary distance.

To clarify the transmission mechanism of NDM-carrying plasmids among *V. parahaemolyticus* strains and their evolutionary relationships with plasmids from different sources, phylogenetic analysis was conducted on *bla*_NDM-1_-carrying plasmids retrieved from public databases via BLAST homology searches. Results demonstrated that the *V. parahaemolyticus* plasmid pVP1434-NDM clustered tightly with four additional *V. parahaemolyticus bla*_NDM-1_ carrying plasmids (pVP148-NDM, pVP156-NDM, pVP205-NDM, pVP209-NDM), forming a well-supported monophyletic clade (Figure 2). Within this clade, pVP148-NDM, pVP156-NDM, pVP205-NDM, and pVP209-NDM were all recovered from shrimp, whereas pVP1434-NDM was isolated from a clinical human specimen. The close genetic relatedness observed between shrimp and human-derived plasmids provides direct molecular evidence supporting the inter-host transmission of *bla*_NDM-1_-carrying plasmids between seafood-associated and clinical *V. parahaemolyticus* strains. All *bla*_NDM-1_-carrying plasmid clades displayed a wide temporal distribution (2016–2024) and geographic diversity, implying the global persistence and dissemination of this plasmid lineage. Notably, the VP-specific clade was phylogenetically distinct from *bla*_NDM-1_-carrying plasmids of other bacterial genera, indicating that these resistance plasmids mainly propagate within the *Vibrio* genus with limited inter-genus transfer.

### 3.6 ST typing and phylogeny

**Figure 3:**
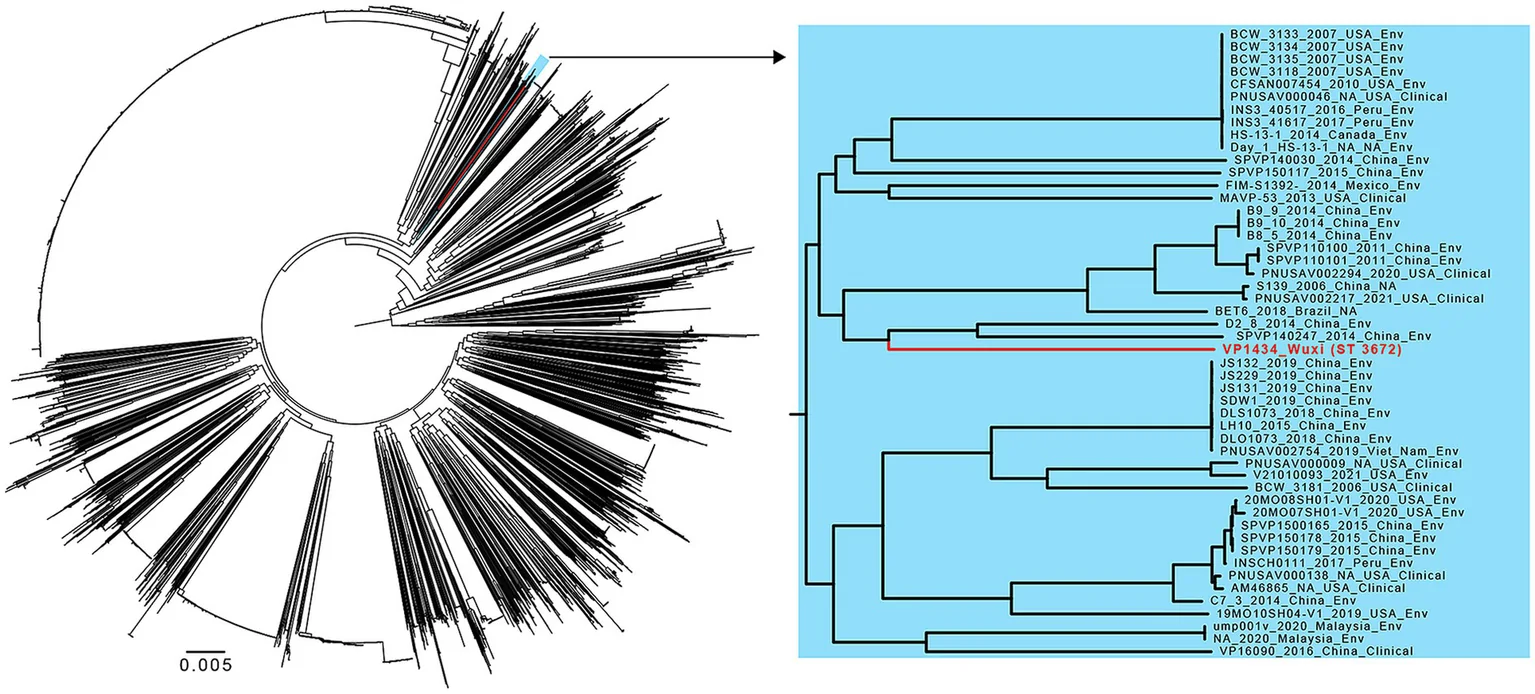
Maximum-likelihood phylogeny of 8,685 global isolates based on core-genome SNPs. Strain VP1434 (highlighted in red) forms a distinct branch, indicating divergent evolution. Scale bar represents 0.05 substitutions per SNP site.

A novel sequence type (ST3672) was assigned by submitting the genome and its allelic profile of housekeeping genes (allelic profile: dnaE:541/gyrB:732/recA:59/dtdS:180/pntA:56/pyrC:615/tnaA:394) to the PubMLST database (https://pubmlst.org/). To investigate the evolutionary relationship between strain VP1434 and other prevalen*t V. parahaemolyticus* isolates, we constructed a maximum-likelihood (ML) phylogenetic tree based on core-genome single-nucleotide polymorphisms (SNPs) (Figure 3). The SNP distance between strain VP1434 and its closest related isolate SPVP140247_2014_China_Env is 49,632 SNPs. According to widely accepted criteria—including a 2,500-SNP cutoff for defining a clonal group, as suggested in a previous study (Yang et al., 2025)—a SNP distance of 49,632 is far larger than 2,500 indicating that VP1434 does not belong to any known clonal group and should be recognized as a singleton forming a unique monophyletic clade. Comparative genomics with global databases confirmed its unique evolutionary position.

### 3.7 Virulence factors in VP1434

Genomic analysis of strain VP1434 screened against the Virulence Factor Database (VFDB) identified 185 virulence factors (VFs) (Table 4). These VFs are mainly linked to core pathogenic processes, including bacterial adherence, antiphagocytosis, chemotaxis, motility, and the assembly and function of specialized secretion systems. Notably, *V. parahaemolyticus* harbors two canonical pathogenicity determinants: thermostable direct hemolysin (*tdh*) and TDH-related hemolysin (*trh*), encoding TDH and TRH toxins, respectively. Initial VFDB screening did not identify either gene, despite this database being a key resource for virulence factor annotation and retrieval. To avoid false negatives and improve detection accuracy, targeted independent BLAST analyses were performed for the *tdh* and *trh* loci. BLAST results showed no homologous *tdh* sequences, confirming the absence of this determinant in VP1434. In contrast, the strain’s *trh* sequence shared 100% nucleotide identity with that of clinical isolate AQ4172 from Japan (accession no: LC271584). Further analysis revealed this *trh* variant is highly divergent from the sole VFDB reference (trhX, VFG044044), explaining the initial detection failure. These results confirm the presence of a functional *trh* virulence gene in strain VP1434.

**Table 4:** Classification and genomic distribution of virulence factors in *V. parahaemolyticus* VP1434.

VF classes	Virulence factors	Predicted genes	No. of genes
Adherence	MSHA type IV pilus	*mshA, mshE--mshN*	11
	Type IV pilus	*pilB--pilD, pilT*	4
	others	*tufA, vpadF, mam7, IlpA*	4
Antiphagocytosis	Capsular polysaccharide	*cpsA--cpsJ*	10
Chemotaxis and motility	Flagella	*cheA, cheB, cheR, cheV, cheW, cheY, cheZ*	7
		*flaA--flaE, flaG, flaI*	7
		*flgA, flgB, flgD, flgF, flgG, flgI, flgJ, flgL, flgN*	9
		*flhB, flhG,flhF*	3
		*fliA, fliF--fliH, fliK, fliL, fliM, fliO, fliS*	9
	Lateral flagella	*flrA--flrC,motB, motX, motY*	6
		*flgC, flgE, flgH, flgK, flgM, flhA, fliD--fliE, fliI--fliJ, fliN, fliP--fliR, lafA, lafS, motA, scrG*	18
		*VP_RS16525, VP_RS16560, VP_RS16575, VP_RS16580, VP_RS16595, VP_RS22390, VP_RS22395, VP_RS22400, VP_RS22495, VP_RS22500, VP_RS22515, VP_RS22520, VP_RS22525, VP_RS22585, VP_RS22590, VP_RS22595, VP_RS22610*	17
Iron uptake	Enterobactin receptors	*irgA, vctA*	2
	Heme receptors	*hutA, hutR*	2
	Vibrioferrin	*pvuA, VP_RS23070, VP_RS23075, VP_RS23080, VP_RS23090, VP_RS23095, VP_RS23100, VP_RS23105, VP_RS230110, VP_RS23115*	11
	ABC transport	*vctC, vctD, vctG, vctP*	4
Quorum sensing	Autoinducer-2	*luxS*	1
	Cholerae autoinducer-1	*cqsA*	1
Secretion system	EPS type II secretion system	*epsC--epsN*	12
	T3SS	*ati2*	1
	T3SS1 secreted effectors	*vopQ, vopR, vopS, VPA0450*	4
	T3SS1	*sycN, tyeA, vcrD, vcrG, vcrH, vcrR, vcrV*	7
		*vecA, virG, vopB, vopD, vopN, vxsC*	6
		*exsA, exsD*	2
		*vscB--vscD, vscF--vscL, vscN--vscU, vscX--vscY*	20
	T3SS2	*vscR2*	1
Toxin	Thermolabile hemolysin	*tlh*	1
	TDH-related hemolysin	*trh*	1
	Hemolysin	*hlyA-hlyD*	4

Although *tdh*^+^ strains cause most clinical *V. parahaemolyticus* infections, sporadic *tdh^−^/ trh^+^* clinical isolates highlight the overlooked virulence of this genotype. Taking the verified functional trh virulence gene into account, strain VP1434 harbors a total of 185 virulence factors. Our findings verify the pathogenicity of VP1434 and emphasize the need for targeted surveillance and detection of *tdh*/*trh* variants in routine pathogen monitoring.

### 3.8 Virulence assessment in murine model

**Figure 4:**
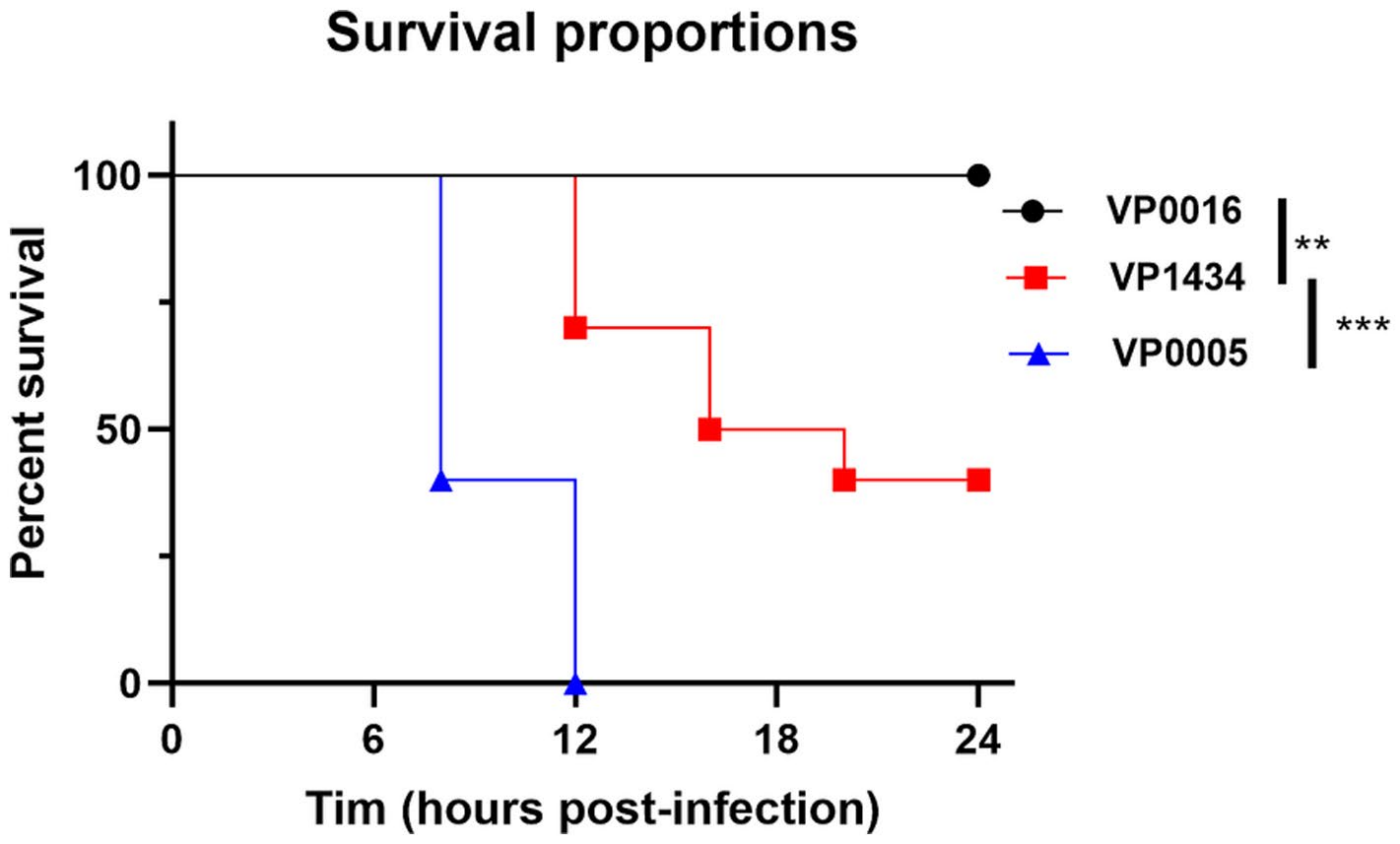
Mice were intraperitoneally challenged with 4.5 × 10^7^ CFU of three distinct strains: VP0016, an environmental isolate lacking both *tdh* and *trh*; VP0005, a clinical strain harboring both *tdh* and *trh*; and VP1434, lacking *tdh* but carrying *trh*. Mortality was recorded at predetermined time points. *p* < 0.01; *p* < 0.001.

To explore whether strain VP1434, which lacks the virulence factors TDH and TRH, retains bacterial virulence and exhibits pathogenicity, we conducted survival experiments in mice. In these experiments, we compared VP1434 against the clinical pathogenic *tdh^+^/trh^+^* strain VP0005 and the environmental *tdh^−^/trh^−^* isolate VP0016. The results demonstrated that although V1434 exhibited lower mortality in mice compared to the common clinical strain VP0005, its virulence was significantly higher than that of the environmental isolate VP0016 (Figure 4). These findings clearly demonstrate that VP1434 maintains pathogenic potential even without the TDH virulence factors, indicating the presence of alternative virulence mechanisms. Notably, extracellular proteases, biofilms, siderophores, and/or high gastrointestinal cytotoxicity may also play significant roles in the pathogenic process.

## 4 Discussion

The MDR strain VP1434, exhibiting a typical virulence and forming an independent branch on the evolutionary tree, poses a significant public health concern that warrants further monitoring. It represents the first documented case of an MDR *V. parahaemolyticus* (VP1434) isolated from human clinical specimens, as such strains have previously only been sporadically detected in the environment and should thus be given serious attention.

The emergence of multidrug resistance in clinical *V. parahaemolyticus* isolates appears to originate from two principal mechanisms: (1) antibiotic-driven selection pressure inducing spontaneous mutations, and (2) horizontal gene transfer (HGT) mediating rapid resistance dissemination (Briet et al., 2018; Li and Zhang, 2022). Antibiotic-driven selection pressure not only selects for pre-existing spontaneous mutations that confer antimicrobial resistance in bacterial populations, favoring the survival and proliferation of resistant bacteria, but also elevates the spontaneous mutation rate by inducing DNA damage and activating stress responses. This process further generates additional novel genetic variations, forming a dual mechanism characterized by “mutagenesis followed by selection”. This dual effect significantly accelerates the evolution and dissemination of bacterial antimicrobial resistance, which serves as a key underpinning mechanism for the formation of MDR bacteria. Notably, such selection pressure is more prone to occur in specific environments, including marine and aquaculture water ecosystems. However, compared with spontaneous mutations, bacteria typically acquire antimicrobial resistance much more rapidly through the gain or loss of resistance-encoding genetic traits. Numerous studies have demonstrated that the emergence of MDR and extensively drug-resistant *Vibrio* spp. as well as other intestinal pathogenic bacteria is mainly attributed to horizontal gene transfer (HGT) mediated by highly dynamic mobile genetic elements (MGEs) (Moura de Sousa et al., 2023). These MGEs include plasmids, integrative and conjugative elements, superintegrons, transposons, and insertion sequences, which can be transmitted among bacteria with either close or distant genetic relationships (Ramamurthy et al., 2020). The natural competence of *Vibrio* spp. enables them to uptake and integrate exogenous DNA from diverse sources, including MDR enteric commensal bacteria, ultimately promoting the emergence of drug-resistant *Vibrio* strains (Dutta et al., 2021). Based on the phylogenetic tree analysis of the pVP1434 plasmid, it is evident that the dissemination of *bla*_NDM-1_ resistance plasmids within the *Vibrio* genus predominantly occurs via intra-genus transmission, with a markedly low incidence of cross-genus transfer events. In sharp contrast, *Klebsiella pneumoniae* and *Escherichia coli* have emerged as pivotal reservoirs for *bla*_NDM-1_ resistance genes. These bacterial species, characterized by their ubiquitous presence in diverse environmental settings, frequently harbor *bla*_NDM-1_ genes on highly mobile plasmids, such as IncFIA and IncX3 (Cai et al., 2026). Such plasmids mediate the rapid interspecies spread of *bla*_NDM-1_ resistance genes, resulting in a significantly higher transmission frequency than that observed within the genus *Vibrio*. This discrepancy may be explained by the specialized ecological niches occupied by *Vibrio* species, which limit their physical interactions with other bacterial taxa. Furthermore, differences in cellular structure and physiological characteristics between genera impede the horizontal transfer of plasmids across genus boundaries.

Globally, *V. parahaemolyticus* resistance to first-line antibiotics such as ciprofloxacin and ceftriaxone is currently on the rise, with China standing out as a major hotspot (Li et al., 2024). Previous molecular characterization has identified several *β*-lactamase genes (*bla*_CMY-2_, *bla*_PER-1_, and *bla*_VEB-2_) in environmental isolates of *V. parahaemolyticus*, which confer resistance to cephalosporins (Zheng et al., 2019). Notably, *bla*_NDM-1_-positive *V. parahaemolyticus* was first detected in environmental samples, such as shrimp, and has now been isolated from clinical patients in the present study. This finding confirms that drug-resistant *V. parahaemolyticus* can transmit from the environment to clinical settings via the seafood chain, underscoring the close link between environmental and clinical antimicrobial resistance. In the present study, the resistant plasmid of strain VP1434 is most probably derived from environmental *V. parahaemolyticus* or other *Vibrio* species. Its conjugative transfer frequency in *V. parahaemolyticus* reached as high as 10^−3^, which may pose a potential threat to clinically prevalent high-virulence *tdh*^+^ *V. parahaemolyticus strains*.

Foodborne diseases caused by *V. parahaemolyticus* strains harboring the *tdh* gene alone or both *tdh* and *trh* genes are significantly more prevalent than those induced by *tdh^−^/trh^+^* isolates. Distinct alleles of the *tdh* gene exhibit extremely high nucleotide homology (> 97%), with two copies located on either plasmids or the chromosome, resulting in low genetic variation. In contrast, the *trh* gene displays remarkable sequence diversity and can be classified into two subtypes, *trh1* and *trh2*, which share only 84% nucleotide homology. Existing studies have demonstrated that all *tdh^−^/trh^+^* clinical and environmental isolates belong to the *trh2* subtype, and the *trh2* sequences of clinical strains show 100% identity to those recovered from clinical isolates in Europe between 1995 and 2008 (Leoni et al., 2016). Sequence polymorphisms in *trh2* can serve as a reliable molecular marker for distinguishing clinical and pathogenic isolates, and research focusing on TRH as a virulence factor would gain greater depth if whole-genome sequencing data from diverse geographical regions and host sources are incorporated. Urease also represents a critical virulence-related factor of *V. parahaemolyticus*. Only a small proportion of clinical *V. parahaemolyticus* strains produce urease, and the *trh* gene is strictly co-localized with the urease gene cluster at adjacent chromosomal loci, making urease production a reliable clinical diagnostic marker for *trh*^+^ virulent strains (Fu et al., 2021). Additionally, currently pandemic pathogenic *V. parahaemolyticus* strains exhibit an evolutionary trend of attenuated virulence (i.e., reduced *tdh* expression) coupled with enhanced adhesion capacity (Yang et al., 2025). This virulence attenuation facilitates bacterial evasion from host immune surveillance and improves colonization and survival within the host, suggesting that *tdh*-associated virulence expression will gradually decline and may even lead to the loss of this virulence gene, while more uncharacterized novel virulence factors are expected to be identified Notably, some clinical strains lacking canonical virulence factors remain pathogenic, indicating the existence of alternative potential virulence determinants and divergent pathogenic strategies among distinct strains (Cai and Zhang, 2018) Animal experiments have also confirmed that *tdh* and *trh* double-knockout strains retain cytotoxicity and intestinal fluid accumulation ability, further corroborating the involvement of other virulence factors in pathogenesis.

*V. parahaemolyticus* encodes two functionally independent Type III Secretion Systems 1 (T3SS1) and 2 (T3SS2), which constitute the core virulence machineries. These two systems act synergistically to regulate host invasion, immune evasion, and the overall pathogenic phenotype, with T3SS1 primarily mediating cytotoxicity and T3SS2, as the core component of the pathogenicity island, governing intestinal infection and enterotoxicity. The vast majority of clinical pathogenic strains harbor an intact T3SS2 gene cluster, whereas strain VP1434 only retains *vscR2*, a T3SS2-associated structural gene whose precise biological function in *V. parahaemolyticus* remains poorly elucidated; it is speculated that this gene plays a pivotal mediating role in human infection. Meanwhile, strain VP1434 harbors an extensive repertoire of T3SS1-related virulence genes. This system is not only associated with bacterial environmental fitness, enabling evasion of host innate immunity, induction of host cell autolysis to release nutrients, and enhancement of stress resistance, but also indirectly modulates early colonization, biofilm formation, motility, and cytotoxicity of the pathogen. *In vitro* cell infection assays reveal that T3SS1 invades and destroys host cells via a cascade of autophagy induction, cell rounding, and eventual lysis, while only perturbing intracellular signaling pathways and preserving nuclear integrity. The key effector proteins of T3SS1 include VepA, VepB, VPA0450, and VopR (Cao et al., 2025), which mediate autophagy initiation, intracellular membrane channel inactivation, cytoskeletal disruption, cell viability impairment, and immunosuppression, respectively. VepA has been shown to be essential for inducing cellular autophagy, and its presence is critical for triggering cytotoxicity (Zakaria et al., 2023); *VepB* targets and inactivates internal cellular channels, leading to cell rounding. VPA0450 is an inositol phosphatase that hydrolyzes phosphatidylinositol phosphates on the plasma membrane, disrupting the interaction between the actin cytoskeleton and the plasma membrane, thereby inducing vesicle formation in the plasma membrane and resulting in cell rounding or lysis. VopR, encoded by its respective gene, can bind to phosphatidylinositol on the host cell membrane, thereby impairing cell viability. Additionally, VopR plays a crucial role in suppressing the proinflammatory immune response during *V. parahaemolyticus* infection, which can facilitate the survival of host cells during infection by *V. parahaemolyticus,* which contains the active T3SS1. Moreover, T3SS1 expression is precisely regulated by the transcriptional activator ExsA (*exsA*) and the anti-activator ExsD (*exsD*), with ExsD directly binding to ExsA to repress the transcription of T3SS1-related genes. Beyond the aforementioned virulence systems, MAM-7 acts as a pivotal virulence determinant that interacts with fibronectin and phosphatidic acid to form a trimolecular complex, further promoting pathogen adhesion to host cells and strengthening the initial colonization capacity of *V. parahaemolyticus*.

Furthermore, the relationship between bacterial virulence and antimicrobial resistance is highly complex and often interdependent. Although relevant reports remain limited in *V. parahaemolyticus*, studies in *Escherichia coli* have demonstrated that quinolone-resistant strains are frequently associated with the loss of virulence factors. Phylogenetic groups with lower virulence may be more prone to acquiring quinolone resistance. Specific uncharacterized genetic features within the *Escherichia coli* genome may underlie such genetic associations (Yang et al., 2021). Similar mechanisms may also exist in *V. parahaemolyticus*, which warrants further investigation.

Several limitations within the scope of this study necessitate explicit clarification. Firstly, the potential for mixed infection in the patient cannot be entirely dismissed, specifically the concurrent presence of both *tdh^+^* and *tdh^−^* strains of *V. parahaemolyticus*. It is well-established that the preponderance of *V. parahaemolyticus* isolates obtained from environmental matrices are avirulent *tdh^−^* variants, whereas clinical isolates are overwhelmingly composed of the more virulent *tdh^+^* counterparts. This disparity underscores the selective enrichment of pathogenic strains within the human host, which confers a competitive advantage to these strains in the *in vivo* milieu. Nevertheless, the limitations inherent in conventional culture-dependent methodologies impede the comprehensive isolation and identification of all extant strains (Wang et al., 2020). In addition, strain VP1434, the *V. parahaemolyticus* isolate obtained in this study, does not belong to pandemic clonal complexes, which somewhat restricts the broader public health relevance and generalizability of the current findings. Considering the close genetic relatedness between foodborne and environmental isolates and non-pandemic pathogenic strains—a characteristic that facilitates horizontal transfer of resistance plasmids—we cannot rule out a stepwise resistance dissemination pattern: resistance plasmids may first reside in non-pandemic foodborne strains, then transfer to non-pandemic pathogenic strains, and eventually spread to pandemic pathogenic clones. This potential transmission pathway provides an important direction for future research.

In conclusion, *V. parahaemolyticus* VP1434 harbors an IncC-type plasmid carrying the *bla_NDM-1_* gene, which endows the strain with multidrug resistance and enables efficient conjugative transfer. This ST3672-type strain exhibits marked pathogenicity despite the absence of *tdh* and represents a distinct evolutionary lineage. Its recovery from a clinical isolate indicates that the *bla_NDM-1_* gene has successfully disseminated among *V. parahaemolyticus* strains from seafood reservoirs to human infections, driving the increasing prevalence of drug-resistant isolates and posing a substantial public health risk. These findings highlight the urgent need for strengthened surveillance and coordinated intervention by public health and food safety authorities.

## Data Availability

The datasets presented in this study can be found in online repositories. The names of the repository/repositories and accession number(s) can be found at: https://nmdc.cn/resource/genomics/genome/detail/NMDC60213730, NMDC60213730.

## Footnotes

1
https://nmdc.cn/resource/genomics/genome/detail/NMDC60213730

2
https://www.ncbi.nlm.nih.gov/nuccore/JBVYWM000000000
