# Correction: Clinical emergence of a novel sequence type (ST3672) NDM-1-producing *Vibrio parahaemolyticus* in foodborne disease

**DOI:** 10.3389/fmicb.2026.1977572

**Published:** 2026-09-08

**Authors:** Chengpei Ni, Jiancheng Wang, Chao Yang, Song Gao, Ze Xu, Yajing Wang, Honghong Peng, Wengting Cao, Yi Han

**Affiliations:** 1 The Affiliated Wuxi Center for Disease Control and Prevention of Nanjing Medical University, Wuxi Center for Disease Control and Prevention, Wuxi, China; 2 Shanghai Institute of Immunology and Infection, Chinese Academy of Science, Shanghai, China

**Keywords:** antibiotic resistance, IncC plasmid, NDM-1, *Vibrio parahaemolyticus*, virulence factors

The figure captions were in the wrong order online version of this paper. The caption published under Figure 2 actually belonged to Figure 3, and the caption published under Figure 3 actually belonged to Figure 2. Importantly, the figures themselves are correct and have not been altered; only the captions were misplaced.

The corrected caption of Figure 2 appears below:

“Figure 2. Phylogenetic Tree of pVP1434-NDM (from VP1434) and other 18 NDM-1-Harboring Plasmids. All plasmid sequences were retrieved from the NCBI public database on February 20, 2026. Sequences obtained from the NCBI GenBank database are indicated by their names; uncharacterized sequences are shown with their accession numbers in parentheses. The tree was constructed using the maximum-likelihood method, with a scale bar of 0.009 indicating the evolutionary distance.”

The corrected caption of Figure 3 appears below:

“Figure 3. Maximum-likelihood phylogeny of 8,685 global isolates based on core-genome SNPs. Strain VP1434 (highlighted in red) forms a distinct branch, indicating divergent evolution. Scale bar represents 0.05 substitutions per SNP site.” The order has now been corrected.

The original version of this article has been updated.

