# Supplementary material for: Clinical emergence of a novel sequence type (ST3672) NDM-1-producing Vibrio parahaemolyticus in foodborne disease
Source: Front Microbiol. 2026 Apr 22;17:1767946. doi: 10.3389/fmicb.2026.1767946 (PMC13144090; doi:10.3389/fmicb.2026.1767946)
Supplement: Supplementary file 1 [file Table_1.DOCX]

Appendix Table 1**.** Codes and Corresponding Test Names for VITEK® 2 GN Card

| **NO.** | **Test Name** | **Full Name** |
| --- | --- | --- |
| 1 | APPA | Alanine-phenylalanine-proline arylamidase |
| 2 | H_2_S | H_2_S production |
| 3 | BGLU | β-Glucosidase |
| 4 | ProA | L-Proline arylamidase |
| 5 | SAC | Sucrose |
| 6 | ILATk | L-Lactate alkalinization |
| 7 | GlyA | Glycine arylamidase |
| 8 | O129R | O/129 resistance (comp.vibrio.) |
| 9 | ADO | Adonitol |
| 10 | BNAG | β-N-Acetylglucosaminidase |
| 11 | dMAL | D-Maltose |
| 12 | LIP | Lipase |
| 13 | dTAG | D-Tagatose |
| 14 | AGLU | α-Glucosidase |
| 15 | ODC | Ornithine decarboxylase |
| 16 | GGAA | Glutamate-glycine-arginine arylamidase |
| 17 | PyrA | L-Pyrrolidonyl arylamidase |
| 18 | AGLTp | Glutamyl arylamidase pNA |
| 19 | dMAN | D-Mannitol |
| 20 | PLE | Palatinose |
| 21 | dTRE | D-Trehalose |
| 22 | SUCT | Succinate alkalinization |
| 23 | LDC | Lysine decarboxylase |
| 24 | IMLTa | L-Malate assimilation |
| 25 | lARL | L-Arabitol |
| 26 | dGLU | D-Glucose |
| 27 | dMNE | D-Mannose |
| 28 | TyrA | Tyrosine arylamidase |
| 29 | CIT | Citrate (sodium) |
| 30 | AGAL | α-Galactosidase |
| 31 | lHISa | L-Histidine assimilation |
| 32 | ELLM | Ellman's reagent |
| 33 | dCEL | D-Cellobiose |
| 34 | GGT | γ-Glutamyltransferase |
| 35 | BXYL | β-Xylosidase |
| 36 | URE | Urease |
| 37 | MNT | Malonate |
| 38 | NAGA | β-N-Acetylgalactosaminidase |
| 39 | CMT | Coumarate |
| 40 | ILATa | L-Lactate assimilation |
| 41 | BGAL | β-Galactosidase |
| 42 | OFF | Fermentation/Glucose |
| 43 | BAlap | β-Alanine arylamidase pNA |
| 44 | dSOR | D-Sorbitol |
| 45 | 5KG | 5-Keto-D-gluconate |
| 46 | PHOS | Phosphatase |
| 47 | BGUR | β-Glucuronidase |
