# Supplementary material for: Clinical emergence of a novel sequence type (ST3672) NDM-1-producing Vibrio parahaemolyticus in foodborne disease
Source: Front Microbiol. 2026 Apr 22;17:1767946. doi: 10.3389/fmicb.2026.1767946 (PMC13144090; doi:10.3389/fmicb.2026.1767946)
Supplement: Supplementary file 2 [file Table_2.DOCX]

| **NO.** | **Drug Name ​** | **​​Abbreviation​** |
| --- | --- | --- |
| 1 | Ciprofloxacin | CIP |
| 2 | Ampicillin | AMP |
| 3 | Ampicillin/Sulbactam | SAM |
| 4 | Amoxicillin/Clavulanic Acid | AMC |
| 5 | Ceftazidime | CAZ |
| 6 | Ceftazidime/Clavulanic Acid | CAZ/CLA |
| 7 | Cefotaxime | CTX |
| 8 | Cefotaxime/Clavulanic Acid | CTX/CLA |
| 9 | Cefoxitin | FOX |
| 10 | Cefepime | FEP |
| 11 | Cefuroxime | CXM |
| 12 | Cefazolin | CZO |
| 13 | Nalidixic Acid | NAL |
| 14 | Florfenicol | FFC |
| 15 | Ceftiofur | CEF |
| 16 | Tetracycline | TET |
| 17 | Tigecycline | TGC |
| 18 | Ertapenem | ETP |
| 19 | Imipenem | IPM |
| 20 | Meropenem | MEM |
| 21 | Ceftazidime/Avibactam | CZA |
| 22 | Polymyxin E | PME |
| 23 | Polymyxin B | PMB |
| 24 | Gentamicin | GEN |
| 25 | Amikacin | AMK |
| 26 | Chloramphenicol | CHL |
| 27 | Azithromycin | AZM |
| 28 | Cotrimoxazole | SXT |
| 29 | Streptomycin | STR |

Appendix Table 2. Antimicrobial Agents for Susceptibility Testing (AST) and Their Abbreviations​
